# The Operation Theatre

**Published:** 1909-10-09

**Authors:** 


### PRACTICAL DEPARTMENTS.

### THE OPERATION THEATRE.

### II.—ITS CONSTITUENT PARTS.

Floor.—The inside of the theatre must be so constructed that it can be hosed down in every part. To this end it is essential that the floor must be so formed as to present an absolutely impervious surface. The substructure or floor proper should be formed of steel joists or concrete, or of one of the many forms of reinforced concrete. The conditions that we have laid down obviously exclude any form of wood floor. Materials other than wood comprise cement, marble terrazzo, tiles, the different forms of plastic floor material now available, and interlocking rubber tiling. Of these the first two—cement and marble terrazzo—must be ruled out on account of their tendency to crack. It is almost impossible to avoid cracking either in cement or in terrazzo, and the cause is probably the same in both cases—that is the expansion and contraction of the cement itself. Tiles, which are largely used abroad, are in themselves a suitable material so far as the texture is concerned, but the very best tiles are not and cannot be made absolutely true and even, and the joints are also an objectionable feature. We are forced, therefore, to the conclusion that the only suitable materials at present available are the plastic floor and the interlocking rubber tile. The plastic floor, of which there are many forms in the market under different names, but of much the same material, is mainly a compound of finely ground sawdust, or wood flour as it is called, and a cementing material, which is usually chloride of magnesia. The floor is laid either in one or two layers, and when laid it forms a perfectly smooth homogeneous surface without any joints whatever. The tendency to crack which was discerned when these floors were first laid in this country has been successfully obviated, and if only the question of colour can be as successfully grappled with these floors have a very great future before them not only for operation theatres, but for all sorts of other purposes in hospitals; and, further, a very strong point in their favour is that they are, compared with terrazzo or tiles, comparatively cheap. The other floor which may be considered a suitable one is the interlocking rubber tiling. This floor consists of solid rubber tiles ⅝ in. thick, which is laid direct on the concrete and has the merit of improving and consolidating as time goes on. Its capacity for wear, is apparently unlimited; but it is a very costly floor, the price being almost nine times as much as that of a good plastic floor.

It is, of course, essential that the floor surface should be laid to a slight gradient, with a channel to carry off the water, and great care is needed in setting out the finished surface so that a level bed is maintained for the operating table.

Walls.—For the wall surface the same condition of imperviousness must be assured. Painted and enamelled cement is probably as good a surface as necessary; but it will not last for ever, and needs periodical renewal. Enamelled tiles are objectionable because of the impossibility of getting very close joints or of ensuring a true surface. Opalite or glass tiles can be fixed with very fine joints, and with reasonable care can be made to present a true and even surface. Slabs of marble of large size have been used, and in some instances alabaster; the great cost of either material surely is not justified by the reduction in the area of joints.

Roof.—The roof of the theatre will be partly opaque, partly of glass. The condition of lighting to be aimed at in the theatre is as nearly as possible open-air; that is to say, a large section of the sloping roof and also of the vertical wall must be of glass. In a small theatre where no accommodation has to be provided for students this is quite easy to accomplish, assuming, of course, that the theatre faces north, as it should; a vertical window about 8 feet wide, carried up to the angle at which it meets the roof and from thence a skylight of the same width and not less than 6 feet long will provide the most perfect light that can be got. The necessary framework should be of light steel and not of wood. The vertical light, though a very great advantage, is not absolutely essential, and in a theatre where stands for students have to be arranged it will generally be found impossible to provide a vertical light. As will be seen when we come to discuss the question of the planning of the theatre and its appurtenances, the entrance for the students must be outside the theatre and not from the theatre corridor. An excellent example in point is the arrangement of the newest theatres at St. Thomas's Hospital. There is no absolutely vertical light, but the provision of skylight is such that the theatre is flooded with daylight. The ceilings or roof in that part not occupied by the skylight should be treated as to their surface in the same way as the walls when either enamel paint, or opalite tiles are used; with any other kind of wall surface the roof or ceiling must be treated with enamel paint.

Doors.—The doors leading into the theatre must have plain flush surfaces with neither mouldings or panels, and must be of hard polished wood. The solid doors made by the Gilmour Door Company are quite the best for the purpose and much less expensive than ordinary solid doors. In many foreign hospitals iron doors have been adopted, but they cannot be said to be a success. They do not fit well as a rule, and are heavy and noisy in use.

Fittings.—The fittings of the theatre itself will comprise glass shelves for lotions and to hold trays, porringers, basins, and other things in use; possibly a fixed lavatory basin and also possibly a slop sink. The slop sink, wherever it is fixed, should be a simple form of white glazed earthenware with a flushing rim and provided with a flushing cistern, which should also be of white glazed ware. The sink should be properly trapped, and the waste pipe must be treated in exactly the same way as a soil pipe. If it is decided to have a fixed lavatory basin with a waste pipe the question will at once arise as to how the taps and the waste are to be worked. There are two alternatives—the treadle or the elbow action. In both the object is the same; that is, that the person using the basin should not be obliged to touch the taps with his hands. Opinions vary greatly on the relative merits of these two systems. It has always seemed to us that the treadle action is superior to the elbow taps, inasmuch as both arms are entirely free, the flow of water being regulated by the foot only. The point is one which suggests discussion, and we should be glad to have the views of experienced readers on it.

